# The effects of antibiotics on the microbiome throughout development and alternative approaches for therapeutic modulation

**DOI:** 10.1186/s13073-016-0294-z

**Published:** 2016-04-13

**Authors:** Amy Langdon, Nathan Crook, Gautam Dantas

**Affiliations:** Center for Genome Sciences, Washington University School of Medicine, Campus Box 8510, 4515 McKinley Research Building, St. Louis, MO 63108 USA; Clinical Research Training Center, Washington University School of Medicine, Campus Box 8051, 660 South Euclid Avenue, St. Louis, MO 63110-1093 USA; Department of Pathology & Immunology, Washington University School of Medicine, Campus Box 8118, 660 South Euclid Ave, St. Louis, MO 63110 USA; Department of Biomedical Engineering, Washington University in Saint Louis, Campus Box 1097, 1 Brookings Drive, Saint Louis, MO 63130 USA; Department of Molecular Microbiology, Washington University School of Medicine, Campus Box 8230, 660 S. Euclid Ave, St. Louis, MO 63110 USA

## Abstract

The widespread use of antibiotics in the past 80 years has saved millions of human lives, facilitated technological progress and killed incalculable numbers of microbes, both pathogenic and commensal. Human-associated microbes perform an array of important functions, and we are now just beginning to understand the ways in which antibiotics have reshaped their ecology and the functional consequences of these changes. Mounting evidence shows that antibiotics influence the function of the immune system, our ability to resist infection, and our capacity for processing food. Therefore, it is now more important than ever to revisit how we use antibiotics. This review summarizes current research on the short-term and long-term consequences of antibiotic use on the human microbiome, from early life to adulthood, and its effect on diseases such as malnutrition, obesity, diabetes, and *Clostridium difficile* infection. Motivated by the consequences of inappropriate antibiotic use, we explore recent progress in the development of antivirulence approaches for resisting infection while minimizing resistance to therapy. We close the article by discussing probiotics and fecal microbiota transplants, which promise to restore the microbiota after damage of the microbiome. Together, the results of studies in this field emphasize the importance of developing a mechanistic understanding of gut ecology to enable the development of new therapeutic strategies and to rationally limit the use of antibiotic compounds.

## Collateral harm from the use of antibiotics

The beneficial impact that the control of bacterial pathogens has had on our standard of living is difficult to overstate. However, our control over microbial disease is diminishing. Human pathogens have repeatedly acquired the genetic capacity to survive antibiotic treatment owing to heavy selective pressures resulting from widespread antibiotic use. The incidence of antibiotic-resistant infections is rising sharply, while the rate of discovery of new antibiotics is slowing, in such a way that the number of withdrawals of antibiotics from healthcare exceeds the number of approvals by a factor of two [1]. In 2015, antibiotic-resistant pathogens were estimated to cause over 50,000 deaths a year in Europe and the USA. The toll is projected to rise to 10 million deaths per year worldwide by 2050 [2]. These figures suggest we are reaching the end of the antibiotic era.

In addition to the development of resistance, the use of antibiotics heavily disrupts the ecology of the human microbiome (i.e., the collection of cells, genes, and metabolites from the bacteria, eukaryotes, and viruses that inhabit the human body). A dysbiotic microbiome may not perform vital functions such as nutrient supply, vitamin production, and protection from pathogens [3]. Dysbiosis of the microbiome has been associated with a large number of health problems and causally implicated in metabolic, immunological, and developmental disorders, as well as susceptibility to development of infectious diseases [4–11]. The wide variety of systems involved in these diseases provides ample cause for concern over the unintentional consequences of antibiotic use. This review will discuss current understanding of these additional effects of antibiotics on the human microbiome, the resulting effects on health, and alternative therapeutic approaches.

## Approaches for identifying a dysbiotic microbiota

It is becoming increasingly apparent that there exist several disease states for which a single causative pathogen has not been established. Rather, such diseases may be due to the abundances and relative amounts of a collection of microbes. Massively parallel sequencing technologies enable quick taxonomical surveys of an entire community by sampling genes from bacterial 16S ribosomal DNA. In addition, to assess functional capability (i.e., the abundances and diversity of metabolic pathways or resistance genes), new computational tools can now analyze short reads from whole-metagenome shotgun sequencing, neatly sidestepping the challenges of read assembly from a complex and uncultured community [12–14]. These methods have been used extensively to establish baseline healthy microbiome compositions, which can then be statistically compared with samples from patients with a disease phenotype. In addition, machine learning algorithms such as random forests can be trained to discriminate between samples from healthy and dysbiotic microbiomes of individuals with a variety of health conditions. This approach ranks taxa in order of discriminatory power and outputs a predictive model capable of categorizing new microbiome samples as either healthy or diseased. Machine learning has been applied to discover which species are important to normal microbiome maturation [15], to malnutrition [16], to protection against cholera [17], and even to development of colon cancer [18]. In addition to high-throughput analysis of gene content, the use of metatranscriptomics [19], metaproteomics [20], and metametabolomics [21] to gain additional insight into the state of the microbiome in various disease contexts has been the focus of increasing interest. These applications underscore the importance of an ecosystem-level view of the gut microbiota in the context of disease diagnosis and therapeutic development.

## The effect of antibiotics on the microbiome in health and disease

### Development and maturation of the microbiome

**Fig. 1:**
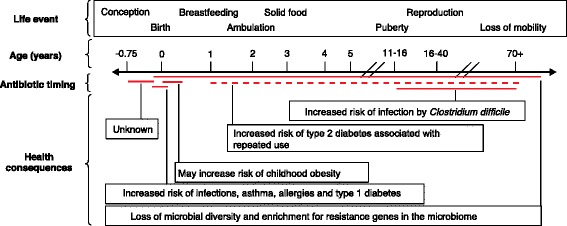
Health consequences linked to the disruption of human-associated microbiota involving antibiotic use during development and adulthood. *Red lines* indicate that a single dose of antibiotics within the time period has been linked to a health consequence, whereas a *dotted red line* indicates that multiple doses of antibiotics within the time period are required to observe a link

As a child grows, the commensal microbiota develops in a predictable succession of species that is generalizable across human populations [15]. The developing bacteriome, the bacterial component of the microbiome, has been profiled many times, both taxonomically and in terms of metabolic functions [15, 22, 23]. These profiles have provided a view of how bacterial species are structured over time. Less is known about the gut-associated eukaryotes and viruses that develop along with the bacteriome, although they are an important part of the gut ecosystem [24, 25]. The disruption of the bacterial succession can be pathogenic [4–7]. Critical developmental milestones for the microbiota (as well as for the child) occur, in particular, during infancy and early childhood, and both medical intervention and lack of such intervention during these periods can have lifelong consequences in the composition and function of the gut ecosystem (Fig. 1). In this section, we discuss the instances in which antibiotics are often used during development and adulthood, the effects of antibiotics on the microbiota, and the implications of such effects for health and disease.

#### Birth

A child’s first contact with microbes is usually assumed to occur after the rupture of the sterile amniotic sac. However, the placenta and the first stool of infants have been found to contain a full complement of microbes [26, 27] and a labeled strain of *Enterococcus faecium* has been shown to cross the umbilical cord in mice [28, 29]. These findings indicate that the first human–microbial interaction occurs before birth, although the effects of this interaction are unknown. Elucidating the function of a prenatal microbiome is especially important; for example, the majority of women in the USA are prescribed antibiotics during pregnancy and delivery [30] and at least 11 types of broad-spectrum antibiotics cross the placenta and reach the fetus [31].

Although the effects of prenatal antibiotics on neonates remain unclear, the microbes that first colonize a child after birth are known to have a fundamental influence on the development of the microbiome. An infant’s mode of delivery is a critical determinant of the composition of their gut microbiota. During vaginal delivery, infants are colonized by the mothers’ vaginal microflora (which is largely composed of *Lactobacillus*, Prevotella, and *Sneathia* species), whereas a Caesarean delivery omits transmission of vaginal microbes. Instead, the first microbes colonizing an infant delivered by Caesarean section are of environmental origin and generally associated with the skin (such as *Staphylococcus*, *Corynebacterium*, and *Propionibacterium* species) [32]. Intestinal strains of *Bifidobacterium* spp. have been shown to be transmitted vertically with vaginal but not Caesarean delivery [33]. Antibiotics are also routinely administered perinatally during Caesarian sections, which is a confounder in these analyses, although it is possible to delay the use of antibiotics until after umbilical clamping, thus separating the effect of antibiotics used by the mother from the effects of those used by the infant. The effects of perinatal administration of antibiotics are likely to further distinguish the microbiota composition of infants delivered by Caesarian section from that of infants delivered vaginally. Postnatal antibiotics can also irreversibly disrupt the natural microbiome succession, as an infant is unlikely to be recolonized with a second dose of vaginal microbes. The composition of the gut microbiome of infants born by Caesarean section has been directly linked with increased susceptibility to, and frequency of infection by, methicillin-resistant *Staphylococcus aureus* (MRSA) [34], which is a symptom of instability and low diversity in the gut ecosystem. Caesarean sections are also associated with a variety of long-term health problems, especially immunological disorders such as asthma [35] and type 1 diabetes [36, 37]. Therefore, elucidating the relationships between these disorders and the composition of the gut microbiome is critical to understanding the risks associated with antibiotic intervention in infants.

Premature birth (birth at <33 weeks of gestation) also has a major influence on the gut microbiome and results in a much greater prevalence of *Proteobacteria* than that usually seen in the *Firmicute*-dominated microbiota of infants born at full term [38]. This trend is aggravated by the aggressive regimen of broad-spectrum antibiotics given to premature infants (generally ampicillin and gentamicin), whose frequency and dosage is usually limited only by the toxicity of the drugs being used (Table 1). Extended antibiotic treatment (>5 days) in premature infants is associated with an increased risk of late-onset sepsis (primarily caused by group B *Streptoccoccus*), necrotizing enterocolitis, and overall mortality [39, 40]. Antibiotic use further shifts the composition of the gut microbiota toward an increased abundance of *Proteobacteria* by depressing *Bifidobacterium* populations [41]. More generally, bacteriocidal drugs decrease the overall diversity of the infants’ gut microbiota and select for drug-resistant microbes [42, 43]. Alternative strategies are needed to prevent and treat infections in premature infants.

**Table 1:** Main antibiotics used for pediatric or adult infections that modify the microbiome

Antibiotic	Molecular target	Class	Resistance mechanism	Effect on gut microbiota	Effect on gut transcriptome	Effect on gut proteome	Effect on gut metabolome
Amoxicillin	Transpeptidase	β-lactam	Altered target, β-lactamase	Reduced abundance enterobacteria [167]	NA	NA	NA
Ampicillin	Transpeptidase	β-lactam	Altered target, β-lactamase	Decreased bacterial diversity, greater prevalence of *Enterobacter* spp. [42]	Increased expression of genes involved in tRNA biosynthesis, translation, vitamin biosynthesis, phosphate transport, stress response, proton motive force, antibiotic resistance and phage [72]; reduced immune cell and mitochondrial gene expression [19]	Increased bacterial glycosidase and mucinase activity [168]	NA
Cefotaxime	Transpeptidase	β-lactam (third generation cephalosporin)	Altered target	Decreased bacterial cell count [169]; decreased abundance of anaerobes and enterobacteria [170]	NA	NA	NA
Chloramphenicol	NA	NA	NA	NA	Increased expression of genes involved in tRNA biosynthesis, translation, vitamin biosynthesis, phosphate transport, stress response, proton motive force, antibiotic resistance and phage [72]	NA	NA
Ciprofloxacin	DNA gyrase	Fluoroquinolone	Altered target, efflux	Decreased abundance of enterobacteria [171]. Lower bacterial diversity [68, 69], decrease in short-chain fatty acid (SCFA) producers [71]	Increased expression of genes involved in tRNA biosynthesis, translation, vitamin biosynthesis, phosphate transport, stress response, proton motive force, antibiotic resistance and phage [72]	NA	NA
Clarithromycin plus metronidazole	Bacterial 50S rRNA/DNA synthesis	Macrolide (clarithromycin) and nitroimidazole (metronidazole)	Altered target/drug inactivation (clarithromycin) and efflux (metronidazole)	Reduction in abundance of Actinobacteria, partial recovery of pretreatment state [70]	NA	NA	NA
Clindamycin	Bacterial 50S rRNA	Lincosamide	Altered target	Initial decreased abundance of enterococci, streptococci, and anaerobic bacteria, subsequent recovery of abundance of streptococci and anaerobic bacteria [172]; reduced diversity of *Bacteroides* spp. [74]; decrease in abundance of bacteria producing short-chain fatty acids [71]	NA	Increased production of immunoglobulin proteins, transthyretin and chymotrypsin-like elastase family proteins; decreased production of proteins involved in T-cell activation, chymotrypsinogen B, phospholipase A2, myosin-1a and cytochrome C [20]	Increased creatine and creatinine, and levels of primary bile acids, N-acetylated amino acids, proline-hydroxyproline, pyroglutamylglutamine, myo-inositol, chiroinositol, methyl-chiro-inositol and γ-glutamyl amino acids, and increased host tryptophan metabolism; decreased levels of secondary bile acids, enterolactone, equol, N-acetyl-aspartate, short-chain fatty acids and sugar alcohols, and decreased bacterial tryptophan metabolism [84]
Erythromycin	Translation	Macrolide	Efflux	Decreases in abundance of Streptococci, enterococci, and enterobacteria; increases in abundance of staphylococci; alteration in abundance of anaerobes [173]	Increased expression of genes involved in tRNA biosynthesis, translation, vitamin biosynthesis, phosphate transport, stress response, proton motive force, antibiotic resistance, and phage [72]	NA	NA
Gentamicin	Bacterial 30S ribosome	Aminoglycoside	Decreased uptake, drug modification	Decreased bacterial diversity, greater prevalence of *Enterobacter* spp. [42]	NA	NA	Increased levels ofoligosaccharides and secondary bile acids; decreased levels of short-chain fatty acids, phenolic acids, uracil, primary bile acids, branched-chain amino acids and aromatic amino acids [85]
Meropenem	Transpeptidase	Carbapenem	Altered target, β-lactamase	Reduced abundance of enterobacteria, streptococci, Clostridia, *Bacteroides* spp., and Gram-negative cocci [174]	NA	NA	NA
Streptomycin	Bacterial 30S ribosome	Aminoglycoside	Decreased uptake, drug modification	Overall diversity decreases; abundance of Ruminococcaceae and Bacteroidaceae increases [20]	NA	Increased production of peptidases, proteins involved in actin polymerization, transthyretin, chymotrypsin-like elastase family proteins, myosin-1a, and cytochrome C; decreased production of chymotrypsinogen B and phospholipase A2 [20]	Bile acid metabolism, steroid metabolism, and eicosanoid synthesis affected; levels of leukotriene B_4_ decrease [88]
Ticarcillin	Transpeptidase	β-lactam	Altered target, β-lactamase	Decreased abundance of enterococci [175]	NA	NA	NA
Tigecycline	Bacterial 30S ribosome	Tetracycline	Altered target, efflux	Reduction in abundance of enterococci, *E. coli*, lactobacilli, and bifidobacteria and increases in other enterobacteria and yeasts [176]; reduction in abundance of Bacteroidetes and increases in Proteobacteria [81]	NA	NA	NA
Vancomycin	Peptidoglycan	Glycopeptide	Altered peptidoglycan target	Decreased bacterial diversity [177]	Increased expression of genes involved in tRNA biosynthesis, translation, vitamin biosynthesis, phosphate transport, stress response, proton motive force, antibiotic resistance, and phage [72]; reduced immune cell and mitochondrial gene expression [19]	NA	Leukotriene B_4_ affected [88]; increased levels of oligosaccharides and decreased levels of short-chain fatty acids and uracil [86]; low doses increase levels of short-chain fatty acids [53]

*NA* data not available

#### Early childhood

The effects of antibiotics on microbial succession, diversity, and resistance can last long past infancy. In the first two or three years of life, a healthy child’s microbiome increases in diversity to resemble an adult microbiome [15]. Bacteriophage (phage) titers start high and drop over time, while eukaryotic viruses are acquired from the environment and accumulate [24]. During this period, microbes are continuously obtained from breast milk, other food, and the environment [44]. When the developmental trajectory of the microbiome is altered by modifying factors, the digestive function can be negatively affected, which can result in either undernutrition or obesity. These phenotypes are often found in underdeveloped and developed countries, respectively. The undesirable microbiome configurations associated with undernutrition and obesity are shaped via selection by diet (calorie restriction or a high-calorie, low-quality diet, respectively) [45], by exposure to disease (high frequency of diarrhea or excessive hygiene) [46], and by the use of medications such as antibacterial agents [47].

Severe calorie restriction during the first years of life has devastating long-term consequences, including damage to learning ability, physical stunting, and diminished economic productivity in the survivors [48]. Undernutrition has a distinct microbial signature consistent with a delay in developmental progression of the microbiome. In Bangladesh, this signature consists of a delay of maturation, which is typically characterized by lower abundances of *Bifidobacterium longum* and increased abundances of *Faecalibacterium prasunitzii*, *Lactobacillus ruminis*, and *Dorea longicatena* [16]. This immature microbiome state is associated with inefficient nutrient extraction from food and vulnerability to enteric infections, which perpetuate the malnourished state and often make nutritional therapy ineffective [49]. Intriguingly, a week-long course of either amoxicillin or cefdinir has been found to improve nutritional recovery and reduce mortality associated with severe acute malnutrition [50]. The combination of antibiotics and nutritional therapy has become standard of care in outpatient management of severe acute malnutrition [51]. The growth response of malnourished patients to therapeutic-dose antibiotics parallels the phenomenon where increased growth is observed in animals given continuous, low-dose, broad-spectrum antibiotics [52]. This effect, as well as more subtle metabolic shifts toward adiposity, has been reproduced in mice [53]. Children from low-income countries also show increased weight gain after antibiotic therapy even when they are not clinically malnourished [54]. More research is needed to establish the mechanisms underlying this treatment and to quantify its repercussions in terms of antibiotic resistance.

On the other hand, obesity has grown to epidemic proportions in developed countries. In 2015, over 30 % of adults and 17 % of children in the USA were estimated to have obesity [55, 56]. The contributions of diet and lifestyle to weight gain are well publicized, but the role of the gut microbes has only recently come to light. A high-calorie diet shifts the microbial ecology toward Firmicutes at the expense of Bacteroidetes, thus increasing the energy harvesting capacity of the microbiota [57]. Microbes from obesity-discordant twins can reproduce the respective phenotypes in gnotobiotic mice [58, 59], which indicates a causal role for the microbiota in obesity. Antibiotic exposure during infancy has been found to increase the risk of overweight in preadolescence for boys [47], although this association was not found in a different population. Similarly, the risk of developing type 2 diabetes increases with repeated use of penicillins, macrolides, cephalosporins, and quinolones [60, 61]. This association could be confounded by the increased susceptibility of people with diabetes to infections requiring antibiotic treatment; however, this possibility is countered by the fact that antifungals and antivirals, which are also more frequently sought by these patients, do not increase the risk of developing diabetes [61]. These findings support the notion that the bacteriome has a strong but uncharacterized role in metabolic disease. Further research is critical to understand the mechanisms underlying these nutritional and metabolic health effects of the bacteriome. This understanding will promote rational and frugal antibiotic use to prevent microbiome disruption and enable the restoration of the microbiota after antibiotic use.

### Adulthood

The mature adult microbiome has been assessed across many populations. The largest project in this area to date is the Human Microbiome Project, which assessed 15–18 body sites in 242 participants in 2012 and continues to sample new individuals [62]. An important finding from this project was that microbial populations differ substantially among healthy individuals, and so far no single microbial composition has been defined as healthy, aside from a preponderance of Bacteroidetes and Firmicutes. General trends observed in follow-up studies include a decrease in microbiome diversity in developed countries compared with the diversity found in hunter-gatherers or societies with restricted access to Western medicine [63, 64]. This difference is often attributed to the hygiene hypothesis, which in addition to improved cleanliness points to the overuse of antibiotics during infections as causal to a reduced microbiome diversity in developed countries. A large range of antibiotics has indeed been shown to transiently or permanently alter the composition of healthy adult microbiotas, usually via depletion of one or several taxa (Table 1). Importantly, the effects of an antibiotic on a microbial community in vivo are likely to be depend on the phylogenetic composition of the community and are not predictable on the basis of the susceptibilities of isolated members of the community to antibiotics observed in vitro. Predicting the effects of antibiotics is complicated by the widely varying concentration of the drug across the body, different microbial growth stages [65], antibiotic-associated induction of phages, interdependence among microbial taxa, and the existence of “cheaters”, or susceptible microbes that are protected by extracellular resistance enzymes produced by other microbes [66]. Repeated empirical measurements of the effects of an antibiotic on a microbial community are therefore the best way to predict how a particular gut microbiome will respond to a given antibiotic.

Oral amoxicillin exposure caused marked shifts in microbiome composition that lasted approximately 30 days on average and were observed for more than 2 months in some of the treated individuals [67]. Large shifts were also reported during an oral course of ciprofloxacin, with the changes persisting for several weeks; the extent of restoration of the baseline composition of the microbiome was highly subject-dependent [68, 69]. A similar subject-dependence in the composition of the microbiome after antibiotic therapy was also observed with cefprozil [63]. The effect of antibiotics also differs by body site, with the throat and saliva recovering their initial microbial diversity after antibiotic therapy much more quickly than the gut [70, 71]. In addition to their effect on the phylogenetic makeup of the microbiome, antibiotics select for resistance in the surviving gut microbiota by stimulating the expression of antibiotic resistance, stress response, and phage genes [72] (Table 1), as well as by increasing the abundance of the resistance genes themselves [73, 74]. These mobilized resistance genes are a reservoir for drug resistance in pathogens [75].

There are multiple and poorly understood interactions between the microbiome and immune system. Failure to regulate immune responses to benign organisms is a common one. Antibiotics interfere with the interaction between the microbiome and immune system, resulting in immunological disorders [35, 76]; antibiotics also increase the host's susceptibility to pathogens [34, 46, 77, 78] (Table 2). Indeed, antibiotics have been shown to alter the transcriptome and proteome of host tissues [19, 20] (Table 1). Perturbations in the host proteome followed a different timescale than perturbations in the species content of the microbiome, with the streptomycin-altered proteome recovering before the microbiota but the clindamycin-perturbed proteome remaining perturbed after microbiota recovery [20]. In an elegant study by Morgun et al. [19], the effects of antibiotics on the host transcriptome were classified by their major cause. The reduction in the number of bacteria in general caused a decrease in gene expression in immune cells, whereas the presence of antibiotics and a prevalence of antibiotic-resistant bacteria together caused a reduction in mitochondrial gene expression and in the number of mitochondria per cell. Although the ability of antibiotics to affect mitochondria (which is due to the bacterial origin of these organelles) was previously known, the researchers identified the virulence-associated molecular pathways of *Pseudomonas aeruginosa* as important drivers of mitochondrial gene loss and host cell death in this study. These and other findings clearly show that antibiotics, alone and through their effects on the gut microbiota, have important effects on host gene expression.

**Table 2:** Examples of antibiotic-induced changes in microbiota that lead to disease

Feature	Effect of antibiotics	Pathological consequence
Antibiotic resistance	Enrichment for resistance genes and resistant organisms [73]. In some cases, the rates of genetic exchange between microbes increase [178]	Multidrug-resistant tuberculosis. Carbapenem-resistant *Escherichia coli* infection [79]
Vitamin production	Depletion of vitamin-producing bacteria	Broad-spectrum antibiotic use (especially β-lactams with an N-methylthiotetrazole moiety) can cause vitamin K deficiency leading to hypoprothrombinemia and uncontrolled bleeding [179]
Digestion	Changes in the proportions of relevant metabolic functions in the microbiome [180]	Altered efficiency of nutrient extraction from food that can contribute to obesity [45, 59]
Diversity	Reduced number of different microbes [68]	Lower diversity reduces ecological stability and resistance to pathogens. Increased susceptibility to infection and diarrhea [34, 46, 77, 78]
Resilience	Decreased availability of microbes to take over newly open niches	Each course of antibiotic acts on a new ecology. Recovery to a stable state, and to a particular stable state, is highly individual [63]
Immune regulation	Increased inappropriate immune activity	Asthma, allergies and autoimmune diabetes have all been linked to antibiotic use [6, 10, 61]
Composition	Varying effects across taxa and for different durations	See Table 1 [41, 67–69, 72]

The majority of studies investigating the effects of antibiotics on the gut metabolome have been focused on susceptibility to infection, most notably with *Clostridium difficile* and *Salmonella typhimurium*. The number of deaths associated with *C. difficile* infection reaches 14,000 per year [79]. Infected patients receive high-dose, extended-duration treatment with multiple antibiotics, yet nevertheless up to 65 % of patients relapse [80]. Recurrence of *C. difficile-*associated diarrhea is associated with a low-diversity microbiome [77]. Exposure to either clindamycin or tigecycline decreases microbiome diversity and increases susceptibility to *C. difficile* infection [78, 81]. Similarly, streptomycin and vancomycin use has been shown to cause an increased susceptibility to *S. typhimurium* infection [46]. The release of sugars and bile acids due to antibiotic-induced depletion of the metabolic activities of gut commensals has been proposed as a potential mechanism for this effect [82, 83]. These nutrients provide an ecological niche that can be exploited by pathogens*.* Multiple studies in which high-throughput metabolomics was performed on an antibiotic-treated microbiome have shown that high concentrations of antibiotics reduce or eliminate most products of bacterial metabolism (including short-chain fatty acids and secondary bile acids), whereas their precursors (including oligosaccharides, sugar alcohols, and primary bile acids) build up [21, 84–87]. In addition, several compounds of the bile acid, steroid, and tryptophan metabolic pathways were significantly altered by antibiotic treatment [88, 89] (Table 1). These metabolic effects seem to be independent of antibiotic class and rather depend on antibiotic concentration, as subtherapeutic doses of penicillin, vancomycin, penicillin plus vancomycin, or chlortetracycline actually increase the concentration of short-chain fatty acids [53]. Multiple metabolic routes exist for *C. difficile* to exploit following antibiotic treatment. In particular, antibiotics deplete the bile acid-hydroxylating activity of *Clostridium scindens*, which is required for protection against *C. difficile* infection [90]. As an additional mechanism promoting infection, antibiotics may enhance bacterial translocation out of the gut [91]. These findings show that provision of broad-spectrum antibiotics can be counterproductive in the treatment of recalcitrant, antibiotic-resistant infections. Alternative strategies such as fecal microbiota transplants (FMTs), which are discussed below, have been used to treat *C. difficile* with a cure rate higher than 90 % [92].

## Alternative approaches for modulating the gut microbiota

### Targeting pathogens while maintaining a healthy microbiota

The examples highlighted above make it clear that overuse of antibiotics can often have negative effects on the host through collateral damage to commensal microbes. As an alternative to broad-spectrum drugs, the development of narrow-spectrum treatments that specifically reduce the capacity of pathogens to cause disease while leaving commensals unharmed has been the focus of increasing interest. The enormous variety of existing antivirulence strategies is briefly summarized here. A more complete discussion of antivirulence therapeutics can be found elsewhere [93–96].

#### Anti-quorum sensing

Quorum sensing (QS) is the mechanism by which bacteria coordinate behavior as a function of population density. The concentration of a continuously secreted signaling molecule serves as a marker of local population size and virulence programs are upregulated or downregulated as a function of this concentration [97]. QS plays a critical part in the virulence of many pathogens, including *Vibrio cholerae* and *P. aeruginosa* [98]. QS can be pharmacologically inhibited in a variety of ways, including destruction of the QS signal [99], acceleration of turnover of key QS proteins [100–102], and competition with the QS signal for binding to key regulatory proteins [103–105]. However, *P. aeruginosa* variants resistant to such quorum-quenching drugs have been recently identified [106, 107] and development of this resistance is thought to be caused by a selective disadvantage in those bacteria lacking QS machinery, even when an infection is not occurring [108]. These observations underscore the risks of having an anthropocentric view of “virulence” pathways and highlight a need for holistic understanding of the roles of such pathways within the cell to develop robust antivirulence strategies.

#### Anti-toxin production

Toxin production is critical to the virulence of a wide variety of species. Small-molecule inhibitors of *C. difficile* major virulence factor toxin B [109], *Bacillus anthracis* lethal factor [110], *B. anthracis* protective antigen channel [111], and *Escherichia coli* verotoxin [112] have been developed as a countermeasure to the activity of these bacterial toxins. Taking inspiration from the body’s own defense repertoire and the historical use of antisera against bacterial infections [113], antibodies against Shiga [114, 115] and anthrax [116] toxins have also been developed. Small-molecule inhibitors of ToxT, the transcription factor controlling the production of cholera toxin, have been shown to be effective in mouse models, though associated with the development of resistance [117, 118]. Finally, inhibitors of type 2, [119], type 3 [119–125], and type 4 [126] secretion systems have been identified, which collectively inhibit the virulence of *Yersinia pseudotuberculosis*, *Chlamidophila pneumoniae*, *Chlamidia trachomatis*, *Shigella flexneri*, *S. typhimurium*, *E. coli*, and *Brucella* spp. Whether inhibition of toxin production is a stable strategy against virulence is unclear because although toxin producers are at an increased metabolic burden relative to nonproducers when the toxin is ineffective, this environment provides a strong selective pressure for anti-toxin-resistant mutants or even for mutants that overexpress the toxin [108].

#### Other antivirulence strategies

Pilus formation is critical to the adherence of uropathogenic *E. coli* to host cell tissue and several compounds that inhibit pili (pilicides) have been effective against this strain [127–130]. Carotenoid production is important to the removal of host reactive oxygen species by *Staphylococcus aureus* and inhibitors of carotenoid production reduce the virulence of this organism [131]. The production of biofilms is important to the virulence of several pathogens and also interferes with the delivery of antibiotics to their target site. Anti-biofilm compounds, in addition to restricting virulence when used as monotherapy [132], could be used in conjunction with broad-spectrum antibiotics or orthogonal antivirulence therapies. Finally, siderophores facilitate the scavenging of rare iron from the host environment and are therefore critical to the survival of several pathogens, including *P. aeruginosa*. Compounds that inactivate siderophores therefore represent an evolutionarily robust antivirulence strategy [133]. Taken together, antivirulence therapies are a promising alternative to traditional broad-spectrum drugs owing to reduction of potential off-target effects as well as reduction in the number of organisms under pressure to develop resistance, even if the ideal “evolution-proof” therapy has not been found.

#### Restoring or enhancing the microbiota

In contrast to approaches focused on targeting certain members of the gut microbiota, strategies have been developed to prevent enteric infections through the delivery of additional or replacement species to the gut to increase its resilience to infection. These strategies include the use of probiotics, fecal microbiota transplants, and phage therapy.

#### Probiotics

Probiotics are defined as “live microorganisms which when administered in adequate amounts confer a health benefit on the host” [134]. Probiotics are often seen as an approach to restore or improve a dysbiotic microbiota [135] and are an effective treatment for a wide range of gastrointestinal diseases, including *C. difficile* infection [136], antibiotic-associated diarrhea [137–139], and acute infectious diarrhea [140]. *Lactobacillus* species are used as probiotics [141], with *L. salivarius* being effective against *Listeria* infection [142] and *L. reuteri* being preventive against antibiotic-associated diarrhea [143]. In addition, *Bifidobacterium animalis* has been shown to protect against infections in infants [144] and *E. coli* Nissle, in addition to being an effective treatment for Crohn’s disease and inflammatory bowel disease [145], has been shown to reduce enteric counts of multidrug-resistant *E. coli* [146]. Most meta-analyses of probiotic use agree that while probiotics can be effective against a range of gut dysbioses, more specific data are needed to determine which probiotics are best for particular patient groups, especially as extensive inter-individual variation exists in the composition of gut microbiota.

Advances in genetic engineering have fueled a growing interest in augmenting the gut microbiota with engineered strains to expand gut function or resilience beyond what can be achieved by administration of unmodified strains. Engineered *Lactococcus lactis* has been used to express and deliver antimicrobial peptides against *E. faecium*, reducing pathogen counts by 10,000-fold in vitro [147]. Excitingly, a recombinant invasive strain of *L. lactis* was used to transfect host cells with engineered DNA in vivo, which led to stimulation of tuberculosis antigen production in mice [148]. Additionally, “sense and destroy” probiotics, which encode sensors for biomarkers of pathogenic strains, have been developed. Upon detection of a pathogen, these probiotics activate a genetic program to kill their target. Two recent studies engineered probiotics to detect 3-acyl-homoserine lactone (used in QS) to specifically target *P. aeruginosa*. Pathogen killing was mediated by expression of engineered antimicrobial peptides in one instance [149] and by increased motility and expression of biofilm degradation enzymes and antimicrobial peptides in the second [150]. Such “smart” therapeutics promise to reduce the development of resistance and off-target effects by restricting treatment to strains of interest in a time-specific and space-specific manner. However, production of killing compounds is not the only mechanism by which engineered probiotics can ward off infections. Increased understanding of nutrient resource (e.g., carbohydrate) utilization within the gut is enabling the development of strains that can outcompete pathogens when available metabolic niches are colonized [82, 151]. Although substantial challenges regarding the safety, containment, and consumer acceptance of engineered probiotics remain to be fully addressed, the therapeutic potential of probiotics enabled by genetic engineering of the gut microbiome is enormous.

#### Fecal microbiota transplants

For opportunistic, antibiotic-resistant infections such as *C. difficile* infections, alternative therapies to antibiotics are far superior to antibiotic-based approaches [152, 153]. The transfer of fecal microbes from a healthy person to a patient has been used as a remedy for recurrent diarrhea for at least 1700 years [154]. This approach is the most comprehensive and crude form of probiotic therapy, as an entire balanced community is administered at once, without necessarily knowing which components are valuable. Healthy fecal microbes are thought to suppress *C. difficile* blooms through niche competition and, potentially, through the production of yet unidentified growth inhibitors. In the near term, FMTs might become a critical tool to limit the spread of antibiotic resistance and lengthen the time to obsolescence for remaining viable antibiotics. In the future, FMTs might be replaced by defined preparations of their constituent therapeutic factors as detailed knowledge of the ecology of the gut microbiota increases.

#### Phage therapy

In addition to its bacterial inhabitants, the gut contains an equally fascinating viral community that exerts a profound effect on the microbiota and, in turn, on the host. As the natural predators of bacteria, phages were used to treat bacterial infections before the advent of antibiotics, after which the use of phage therapy was restricted to the USSR [155]. As antibiotics have become less effective, phages have been the focus of renewed therapeutic interest as they are often highly specific to their target bacteria (which reduces off-target effects on the rest of the microbiota) and are self-replicating (which reduces the costs of producing phage-based therapeutics relative to the costs of producing small-molecule therapeutics and also enables co-evolution of the therapies and their pathogen targets). Phages active against *E. faecalis* [156], *Bacillus cereus* [157], and *P. aeruginosa* [158] have been identified, among many others. As is the case for antibiotics, the development of resistance to phages is evolutionarily favorable, but phage-resistant mutants have been observed to be less virulent than their phage-susceptible wild type for some bacteria/phage combinations [159, 160]. Excitingly, phages have also been the subject of genetic engineering to improve their function in modulating the gut ecosystem [161]. In particular, the expression of a biofilm-degrading enzyme on the genome of T7 phages enabled simultaneous reduction of biofilm and bacterial lysis in a positive-feedback manner [162]. T7 phages have also been engineered to encode quorum-quenching enzymes as a defense against biofilm formation [163]. Recently, the natural transformation capacity of phages has been coupled with programmable nucleases to enable the generation of phages that specifically kill bacteria with undesirable genomic sequences, such as antibiotic resistance genes or virulence factors [164, 165]. By programming sequences from resistance genes and lytic phages as substrates for nucleases, Yosef et al. [166] generated a system with a positive selective pressure for loss of antibiotic resistance. On the basis of these reports, we envision that the first diseases for which phage therapy would be appropriate are those whose bacterial cause is well-defined, refractory to antibiotics, and accessible to phages, such as diseases caused by *Mycobacterium tuberculosis*, *V. cholerae*, *C. difficile*, enteroaggregative *E. coli*, and diffusely adherent *E. coli*. Although substantial hurdles involving resistance to both phages and engineered nucleases need to be cleared, natural and engineered phages hold great promise as future tools in the fight against pathogens and dysbiotic community states.

## Conclusions and future directions

Antibiotics shape the ecology of the gut microbiome in profound ways, causing lasting changes to developing and mature microbiotas. The application of next-generation sequencing has enabled detailed views of the side effects these drugs have on commensal populations during treatment of infections. In addition to the increased threat of resistance to antibiotics caused by the overuse of these compounds, these important side effects make it clear that overuse of broad-spectrum antibiotics must be quickly phased out in favor of more precise approaches and must be complemented by efficient methods to restore the microbiome after injury. Fortunately, recent advances in the development of narrow-spectrum antivirulence compounds, coupled with a renewed interest in the use of probiotics, FMTs and phage therapy, bring new hope to defeating disease-causing bacteria while limiting collateral damage to the microbiota. Looking ahead, we anticipate that individualized ecological and metabolic models of the microbiome will have an important role in informing treatment options during dysbiosis, and that these treatment options will be expanded to include evolution-resistant antivirulence compounds, robust curated communities of healthy gut commensals, and “smart” living therapeutics that sense and respond to disease states with minimal patient and doctor intervention. Collectively, advancements in our understanding of the effects of antibiotics on gut commensals are leading to new insights into this complex and important microbial community and are driving new therapeutic strategies in our fight against pathogenic bacteria.

## Abbreviations

FMT: fecal microbiota transplant
MRSA: methicillin-resistant *Staphylococcus aureus*
QS: quorum sensing
